# Brain atrophy in multiple sclerosis: mechanisms, clinical relevance and treatment options

**DOI:** 10.1186/s13317-019-0117-5

**Published:** 2019-08-10

**Authors:** Athina Andravizou, Efthimios Dardiotis, Artemios Artemiadis, Maria Sokratous, Vasileios Siokas, Zisis Tsouris, Athina-Maria Aloizou, Ioannis Nikolaidis, Christos Bakirtzis, Georgios Tsivgoulis, Georgia Deretzi, Nikolaos Grigoriadis, Dimitrios P. Bogdanos, Georgios M. Hadjigeorgiou

**Affiliations:** 1 Department of Neurology, Laboratory of Neurogenetics, Faculty of Medicine, University of Thessaly, University Hospital of Larissa, Biopolis, Mezourlo Hill, 41100 Larissa, Greece; 2 Immunogenetics Laboratory, 1st Department of Neurology, Medical School, National and Kapodistrian University of Athens, Aeginition Hospital, Vas. Sophias Ave 72-74, 11528 Athens, Greece; 3 Department of Rheumatology and Clinical Immunology, Faculty of Medicine, School of Health Sciences, University General Hospital of Larissa, University of Thessaly, Viopolis, 40500 Larissa, Greece; 4 Multiple Sclerosis Center, 2nd Department of Neurology, AHEPA University Hospital, Aristotle University of Thessaloniki, Thessaloniki, Greece; 5 Second Department of Neurology, School of Medicine, University of Athens, “Attikon” University Hospital, Athens, Greece; 6 Department of Neurology, Papageorgiou General Hospital, Thessaloniki, Greece; 7 Department of Neurology, Medical School, University of Cyprus, Nicosia, Cyprus

**Keywords:** Multiple sclerosis, Bran, Atrophy, Neurodegeneration, Axon, Inflammation, Neuroprotection, Drugs

## Abstract

Multiple sclerosis (MS) is an immune-mediated disease of the central nervous system characterized by focal or diffuse inflammation, demyelination, axonal loss and neurodegeneration. Brain atrophy can be seen in the earliest stages of MS, progresses faster compared to healthy adults, and is a reliable predictor of future physical and cognitive disability. In addition, it is widely accepted to be a valid, sensitive and reproducible measure of neurodegeneration in MS. Reducing the rate of brain atrophy has only recently been incorporated as a critical endpoint into the clinical trials of new or emerging disease modifying drugs (DMDs) in MS. With the advent of easily accessible neuroimaging softwares along with the accumulating evidence, clinicians may be able to use brain atrophy measures in their everyday clinical practice to monitor disease course and response to DMDs. In this review, we will describe the different mechanisms contributing to brain atrophy, their clinical relevance on disease presentation and course and the effect of current or emergent DMDs on brain atrophy and neuroprotection.

## Introduction

Multiple sclerosis (MS) is an immune-mediated disease that affects the entire central nervous system (CNS) [1–3]. Magnetic resonance imaging (MRI) lesions are well-scattered at white matter (WM) and grey matter (GM) [4], while normal-appearing brain tissue in MRI also seems to be affected in pathological studies [4]. Brain atrophy, the gradual loss of brain volume, is quite extensive in MS, nearly 0.5–1.35% per year, far off the limits of normal aging [5, 6]. It arises early in the course of the disease, accelerates with disease progression [7–12] but is attenuated by disease-modifying drugs [13].

There has been increasing interest in measuring tissue loss in CNS, as it represents the net effect of all destructive pathogenic processes during the disease course [14–17]. It is worth recalling that neurons occupy almost half (46%) of the tissue volume, myelin is 24%, and glial and other cells almost 30% [5]. GM [4] holds much less myelin than WM (about one tenth), while neurons comprise its most abundant component [18]. Relative to glial cells, oligodendrocytes outweigh the number of astrocytes, microglia and oligodendrocyte progenitor cells, although the exact percentage is still unknown [19, 20].

Atrophy in MS is often considered to be the result of extensive axonal transection and demyelination [21–23]. The contribution of neuroglia may be less clear; reactive gliosis has the potential to mask considerable tissue loss in WM lesions [24, 25]. Measurement of brain atrophy is also considerably influenced by the amount of tissue fluids [26], which is increased by active inflammation and vasogenic edema in WM plaques, and decreased during treatment with agents with strong anti-inflammatory properties (pseudoatrophy effect) [14, 26].

Transient volume changes could also be attributed to idiosyncrasic and technical factors [14]. Dehydration may affect functional integrity of neuroglial cells, while decreased protein levels mainly affect synaptic densities [26]. Unlike demyelination, water volume fluctuations and transient biological factors, neuroaxonal damage is irreversible in CNS, and atrophy is primarily considered to reflect this neurodegenerative component in MS [27–30]. Finally, the atrophy rates may also be influenced to some extent by the genetic makeup of a person; Human leukocyte antigen (HLA) genotypes considered as ‘high risk for MS’, namely DRB1 and DQB1, have been associated with significantly lower WM and GM volumes, alongside with higher mean annualized percentage of brain volume change (PBVC) compared with medium and low risk HLA genotypes independent from patients clinical features (age, gender, disease course) or the DMTs used [31].

## Pathogenesis of brain atrophy

### The time trajectory of brain atrophy

Focal tissue loss in WM plaques is undoubtedly a major contributor to brain atrophy. However, the correlation between demyelination foci and whole brain atrophy is still a matter of debate [16]. Some studies have found a strong association [32, 33], while others have not [25, 34–36], suggesting that separate pathologic processes may also contribute to tissue destruction.

Chard et al. [37] in a longitudinal 14-year study found that atrophy is more related to early rather than late focal lesion volumes. Inflammation may be an important contributor to global tissue loss in early disease stages (i.e. in clinically isolated syndrome). As the disease progresses, additional mechanisms emerge that are, at least partly, independent from WM injury, such as microglia activation, meningeal inflammation, iron deposition, oxidative stress and diffuse axonal damage in normal appearing white matter (NAWM). The lack of a significant relationship between white matter fraction (WMF) and T2 lesion load [34, 38] further support this hypothesis. Biopsy studies also confirm that the atrophy may proceed even in the absence of inflammation [39, 40].

Regional atrophy studies may also be helpful. Indeed, the volume loss of deep GM structures may be present in the early stages of the disease and it is strongly correlated with the disease course [41]. In MS, brain atrophy may develop in different CNS structures and varies depending on the clinical disease phenotypes; ventricular enlargement is more prominent in relapsing–remitting MS [RRMS], whereas cortical atrophy seems to be more important in the progressive forms of the disease [42].

All things considered, it has been suggested that the pathogenic trajectory of brain atrophy changes with disease progression; from primarily inflammatory to less inflammatory and primarily neurodegenerative in the late stages of the disease [43, 44].

### Pathogenesis of acute demyelination and axonal injury

In the initial stages of MS, many different components of the adaptive and the innate immunity induce demyelination and neuronal loss [43]. The activation of auto-reactive CD4+ T lymphocytes in the peripheral immune system is necessary for their migration across the blood–brain-barrier (BBB) and into the CNS. After myelin destruction, T cells are in situ reactivated by antigens within myelin debris and their clonal expansion results in multifocal demyelinating plaques [45]. Peripheral B lymphocytes are involved in the antigen presentation and initial stimulation of CD4 T cells. Also, they are an essential source of pro- and anti-inflammatory cytokines (IL-6 among others) promoting every autoimmunity response (driven by Th1, Th2, Th 17 cells) driving MS. In addition, the presence of chemokines (CXCL13) and survival factors (BAFF and APRIL) in the CSF of patients with MS, promotes the formation of meningeal follicle like structures, in progressive phases but also in early RRMS [46]. T cells and B cells may, therefore, play an equally important role in the immunopathology of MS [47].

Axonal destruction is quite extensive (up to 60–80%) in all active WM lesions [9, 12, 48] and the extend of axonal loss is related to the number of immune cells within the plaques [49]. Activated immune cells (T and B cells) and microglia/macrophages release a number of pro-inflammatory cytokines (e.g. TNFa, INFγ), proteolyticenzymes (e.g. perforin, granzymes) and free radicals (e.g. nitric oxide, glutamate) that can directly damage axons [50]. Additionally, axons may die secondarily, due to the loss of pre- and post-synaptic signals (i.e. dying-back and Wallerian axonal degeneration) in regions far from the lesion site [43].

Active MS lesions are characterized by profound heterogeneity regarding their demyelination pattern [51], which is persistent over time [52]. The most commonly observed patterns are pattern II, which is a complement- and antibody-mediated demyelination, and pattern III, in which the initial event in lesion formation is a brief yet exorbitant oligodendrocyte injury [53]. In other patients with RRMS, new lesions are associated with T cells, and activated microglia only. Pathologic heterogeneity across individuals in demyelination may imply different stimuli in the initial inflammation or different vulnerability to tissue loss across individuals [54].

In WM lesions, inflammation and brain edema, demyelination, axonal loss, gliosis, and remyelination, all happen simultaneously [35, 55]. Brain edema which increases brain volume might bias atrophy measurements, but it resolves in the first few weeks after lesion formation. Notably, CNS has the capacity to use a great number of compensatory mechanisms (i.e. remyelination, redistribution of sodium channels, expression of neurotrophic factors etc.) to re-establish lost functioning to demyelinated foci [48].

To conclude, tissue loss due to inflammation and demyelination maybe partly reversible in RRMS [56, 57], while tissue loss and axonal damage due to mechanisms other than inflammation is irreversible, and remains the major component of brain atrophy especially in the progressive disease stages.

### Mechanisms of late axonal loss (Fig. 1)

While the destruction of CNS myelin is associated with clinical relapses, acute or late axonal loss is considered to be the main cause of permanent clinical disability in MS [49]. Axons are more vulnerable to acute injury by inflammatory mediators, due to their shape and structure, compared to cell bodies or dendrites [43], while thin axons (< 2.5 μm in diameter) are mainly affected [24, 58]. Neurofilament light chain (NfL) protein is only expressed in neurons. It is an essential component of the axonal cytoskeleton, and reflects the axonal integrity and the stability of neurons. Under conditions of acute axonal transection, NfL are released and can be found as a result, in the cerebrospinal fluid (CSF) and blood of patients with MS. Of note, ultra high versus low blood NfL levels have been associated with MRI related (increased number of gadolinium enhancing or T2 lesion load, whole brain atrophy) and clinical measures (number of relapses, disability worsening) of disease activity and evolution and may, therefore, have prognostic value for patients and clinicians [59].

**Fig. 1:**
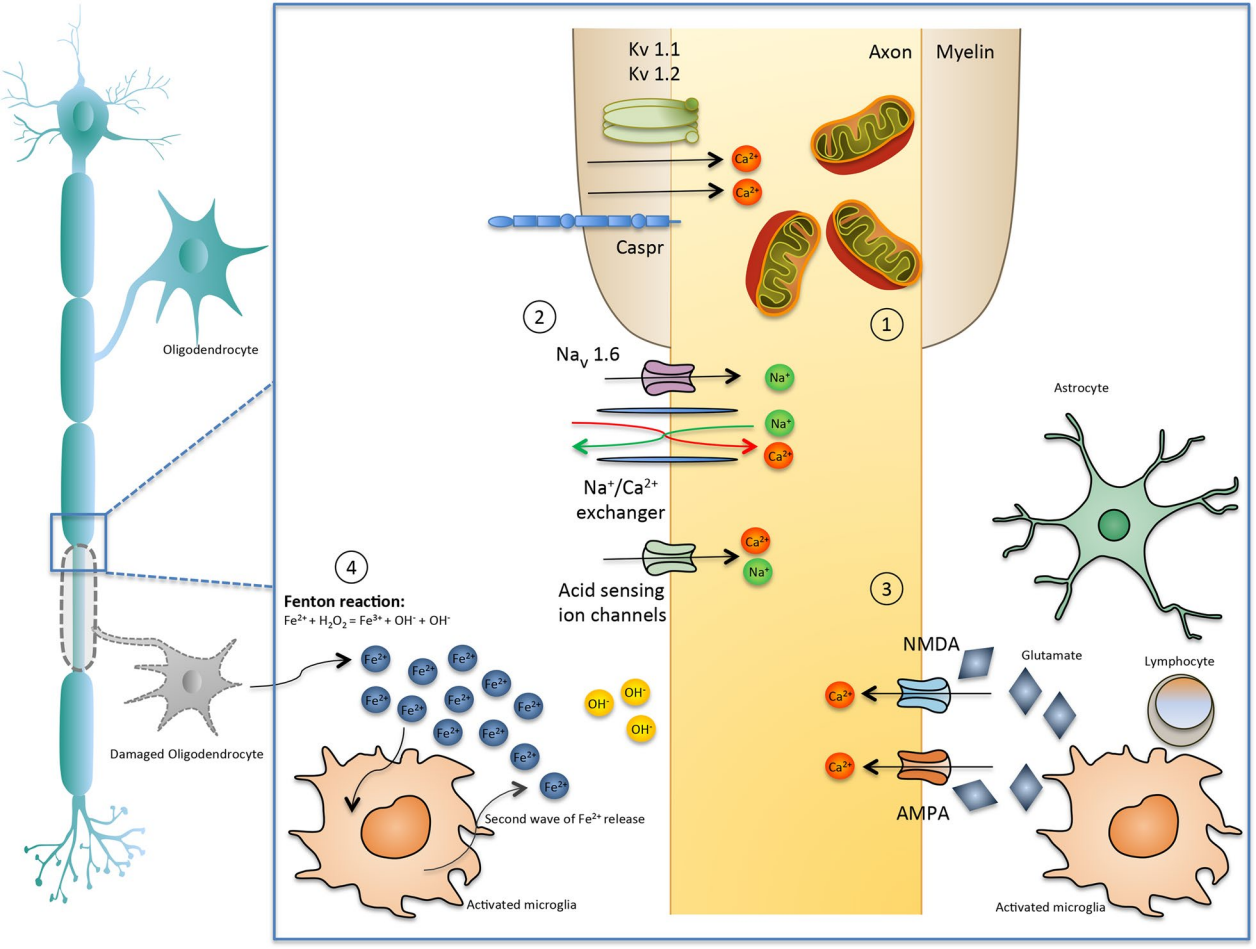
Mechanisms of late axonal loss. Molecular and cellular mechanisms driving neurodegeneration and atrophy. Key elements are considered to be: (1) Mitochondria Dysfunction: Inflammation in acute demyelinating lesions lead to respiratory protein complexes inhibition, mitochondrial injury and dysfunction, release of apoptosis-inducing factors and mitochondrial DNA deletions. In chronic inactive plaques, ionic imbalance, high energy demands and clonal expansion of defective mitochondria further impair oxidative damage. These mitochondrial alterations of functional impairment and structural damage lead to histotoxic hypoxia and energy failure and consequently to neurodegeneration. [146] Upregulation of sodium channels, acid sensing ion channels and expression of maladaptive isoforms (Nav1.6 channels), paranodal (Caspr) and juxtparanodal (Kv1.2) protein lead to high energy demands, intra-axonal calcium accumulation, and subsequent axonal degeneration. (3) Glutamate Excitotoxicity: Increased glutamate production by activated microglial cells and lymphocytes, and impaired clearance by resident cells such as astrocytes lead to higher lever of glutamate. High levels of glutamate lead to over-activation of *N*-methyl-d-aspartate (NMDA) and α-amino-3-hydroxy-5-methyl-4-isoxazolepropionic acid (AMPA) receptors (which are permeable for calcium and sodium ions) and subsequent calcium overload and oligodendocyte and neuron cell death. (4) Iron release: In MS lesions free iron [Fe2+] is released in the extracellular space leading to production of highly reactive hydroxyl molecules (OH^−^) by the Fenton reaction. Further, iron is released by activated glial cells, which become dystrophic and disintegrate, leading to a second wave of Fe^2+^ release

Transected axons and ovoids are abundant in MS lesions [9, 27] but, abnormalities have also been reported in chronic inactive plaques, in normal appearing white matter (NAWM), and cortical areas, in which inflammation is less prominent [48, 57]. Therefore, additional mechanisms of axonal loss coexist with disease progression. It should be noted that these mechanisms have been postulated for both acute and late axonal loss (i.e. “late” signifying the absence of apparent inflammation):

#### Ion overload

Several ion channels show compensatory changes a few weeks after demyelination [60] a process that eventually promotes energy deficiency, and neurodegeneration. Aberrant expression of sodium channels, acid sensing ion channels, increased expression of maladaptive isoforms (i.e. Na_v_1.6 channels) [61], paranodal (Caspr) and juxtparanodal (K_v_1.2) protein alterations [62] have also been detected in WM lesions, in NAWM, and GM. Alternation in the expression of these ion channels lead to intra-axonal calcium accumulation, and subsequent axonal degeneration and atrophy, particularly in secondary progressive MS [49].

#### Mitochondria dysfunction

There has been increasing interest in the role of mitochondrial injury in MS demyelination and axonal destruction. In acute inflammatory lesions mitochondrial nicotinamide adenine dinucleotide-hydrogen (NADH) oxidase [63] and complex IV defects (COX I) have been described, in axons, oligodendrocytes, and astrocytes [58]. In chronic inactive plaques, ionic imbalance and high energy demands result to swollen and dysfunctional mitochondria [64, 65], a phenomenon in which is partially reversed in remyelinating axons [66]. There are also additional mtDNA deletions in GM structures of patients with SPMS [67]. Furthermore, the respiration deficient neurons were diffusely distributed in the subcortical WM resulting in axonal loss in the absence of demyelination or inflammation. In oligodendrocytes, mitochondrial damage results in cell death and demyelination. Progenitor cells are also impaired, regarding their capacity to differentiate and produce myelin [48]. Plus, genetic defects in mitochondrial genes potentiate MS lesions [68]. From what can be deducted, mitochondrial dysfunction, in neurons and glia, is recognized as an important cause of atrophy and degeneration in MS and in other primarily neurodegenerative deceases such as Alzheimer’s disease and Parkinson’s disease [65, 69].

#### Iron dysregulation

Iron [Fe] loading accumulates with age and in patients with MS, it can further increase oxidative tissue loss. In the CNS, iron is mainly stored in oligodendrocytes, binding with ferritin. Under conditions of oxidative stress, such as MS lesions, when oligodendrocytes are destroyed, free iron [Fe^2+^] is released in the extracellular space and becomes an additional source of reactive oxygen species (Fenton reaction: Fe^2+^ + H_2_O_2_ = Fe^3+^ + OH. + OH−) [48]. Further, iron is released by activated glial cells, which become dystrophic and disintegrate, leading to a second wave of Fe^2+^release.

Diffuse T2hypointenselesions, which represent increased iron deposition [70] are commonly found in patients with MS in cortical and deep GM areas (i.e. thalamus, basal ganglia, dentate nucleus [71–73] and WM plaques [74]. Notably, T2 hypointensity has been associated with brain atrophy and early axonal loss [73]. Furthermore, in progressive MS, there is a significant decrease in iron levels in NAWM [75]. Iron is important for myelin synthesis and neurogenesis, and iron depletion in normal appearing tissue, may further promote diffuse axonal loss and CNS atrophy.

#### Glutamate excitotoxicity

Several lines of evidence suggest that glutamate could also mediate injury to myelin, oligodendrocytes and neurons in the autoimmune experimental encephalomyelitis (EAE) model and in MS [76]. Glutamate levels are elevated in CSF [77], in the centre of active plaques, on the borders of chronic active lesions [78], and in NAWM [79].

There are two factors intertwining for glutamate accumulation: increased glutamate production by activated microglial cells and lymphocytes, and impaired clearance by resident cells such as astrocytes. High levels of glutamate lead to the over-activation of *N*-methyl-d-aspartate (NMDA) and α-amino-3-hydroxy-5-methyl-4-isoxazolepropionic acid (AMPA) [80] receptors (permeable for calcium and sodium ions) and subsequent calcium overload and oligodendocyte and neuron cell death.

## Clinical correlates of brain atrophy

Clinical symptoms and signs do not usually correlate with changes seen on conventional MRI measures (the “clinical-MRI paradox”) [81, 82]. Whole brain atrophy, on the other hand, has a significant imaging association with physical disability as measured by Expanded Disability Status Scale (EDSS) score [83–88]. In a longitudinal study, whole brain (WB) and cortical atrophy as well as other MRI related metrics such as the enlargement of ventricular CSF spaces have been associated with disability progression over a 10 year follow up [89]. Furthermore, brain volume changes during the first year after disease onset, estimated by PBVC, were the best predictor of future neurologic impairment [90] regardless of the intermediate relapse rate [91]. Increased brain volume loss (BVL) has been correlated disability progression, independent from the number of previous relapses or the T2 lesion load in RRMS [92].

In a similar vein, when patients with clinically definite MS were compared to patients with clinically isolated syndrome (CIS), at baseline, all brain volume metrics, except for cortical GM, were significantly lower in the MS cohort. Over a mean follow-up period of about 3 years, the annual PBVC values were significantly lower in CIS patients when compared to the MS cohort [93]. Neuropsychological impairment, affecting mental speed processing, episodic memory, executive functions and attention, may be present in up to 50% of patients with MS [94] and has been found to occur early in the disease course [95]. Changes of brain parenchymal fraction (BPF) have been shown to predict cognitive impairment over 2 years in patients with early MS [96]. Cortical atrophy was the best predictor of poor cognitive functioning, even when mild impairment was detected. Poorcognitive functioning has been associated with significant cortical thinning [97], especially in the fronto-parietal cortical and subcortical regions [98]. Pravatà et al. [98] specifically reported that the thinning of the right precuneus and high T2 lesion load were the best predictors of cognitive impairment. Strong correlations have also been reported between cognitive impairment and thalamic atrophy [80, 98, 99]. Not surprisingly, patients with brain atrophy and higher education or high “cognitive reserve” are relatively protected against cognitive decline [100].

Other clinical aspects of CNS atrophy include mood and personality disorders (i.e. euphoria, disinhibition, aggression, major depressive disorder) [101] autonomic dysfunction and sexual disorders [85]. Fatigue has been reported to be associated with GM atrophy in frontal regions [102] and depressed patients were found to present selective cortical thinning in the fronto-temporal regions, while the frontal thinning was found to be the best predictor for depression in MS patients [98].

Taken together, this growing body of evidence suggests that brain atrophy is a valid and sensitive measure of disease burden and progression in MS patients and may effectively be used in routine clinical practice and treatment trials.

## Effect of disease modifying treatments (Tables 1, 2 and 3)

### Approved DMTs and brain volume outcomes

The need of agents to control the inflammatory process in multiple sclerosis pathology is obvious, but the need for medications to halt brain atrophy progression and neurodegeneration is also evident. Currently approved treatments for MS differ in their effects on brain atrophy [103] (Table 1 for the first line therapies, Table 2 for the second line therapies and Table 3 for the emerging therapies).

**Table 1:** First line therapies and their effect on brain volume loss (BVL)

References	DMT and trial design	Clinical trial	Baseline/MRI cohorts	Type of MS	Main effect on brain atrophy
Rudick et al. [83, 87]	IFN β-1a i.m. 30 mcg weekly vs placebo	Phase III MS.C.RG 2 years	140 IFN β-1a n = 68, placebo n = 72	RRMS	Percent change inbrain parenchymal fraction was lower in IFN β-1a treated patients compared to placebo, during the second year of treatment (p = 0.03) but not the first (p = 0.71)
Fisher et al. [104]	IFN β-1a i.m. 30 mcg weekly vs placebo	Retrospective analysis of Phase III MS.C.RG 2 years	131 IFN β-1a n = 62, placebo n = 69	RRMS	IFN β-1a significantly preserved GM [4] atrophy (p = 0.03) and whole brain atrophy (p = 0.04) during the second year of treatment, but not WM atrophy (at any point)
Fillipi et al. [106]	IFN β-1a s.c. 22 μg weekly vs placebo	Phase III ETOMS 2 years	262 IFN β-1a n = 131, placebo n = 132	CIS	Significant reductions in PBVC the IFN β-1a treated arm (p = 0.0031) compared to placebo, from baseline to second year
De Stefano et al. [173]	IFN β-1a s.c. 44mcg TIW vs placebo	double-blind and rater-blind phase IMROVE 40 weeks	180 (double-blind phase) IFN β-1a n = 120, placebo n = 60	RRMS	Non-significant differences in mean [102] PBVC between treatment groups (placebo: − 0.24% [0.48%]; IFN β-1a: − 0.22% [0.54%]; p = 0.76) at week 16 (end of double-blind phase)
De Stefano et al. [105]	IFN β-1a s.c. 44 mg TIW vs once a week vs placebo	Phase III REFLEX 2 years	517 IFN β-1a TIW n = 171, IFN β-1a, once a week n = 175, placebo n = 171	CIS	No differences in BVL (from baseline to 2 years) in patients receiving once or three times a week IFNβ-1a vs placebo. The greatest loss was in the TIW IFN β-1a group compared with the once a week IFN β-1a and placebo groups
Kappos et al. [108]	IFN β-1a s.c. 44 or 22 mcg TIW vs placebo (2 years); then open label (4 years); long term follow up (2 years)	Phase III PRISMS ~ 8 years	382 44 mcg sc TIW n = 136, 22 mcg sc TIW n = 123, placebo n = 123	RRMS	Non-significant differences in median BPV (from baseline to long term follow-up and each study period therein) for all treatment arms
Hardmeier et al. [174]	IFN β-1a i.m. 30 μg or 60 μg	Retrospective of The European IFNb-1a Dose- Comparison Study 3 years	annual MRI cohort n = 386, frequent MRI cohort n = 138	RRMS	The greatest BVL took place during the first 4 months of therapy in frequent MRI cohort (from baseline to month 4, p < 0.05). Non-significant reduction in the brain atrophy in the 2nd and 3rd year of treatment
Molyneux et al. [175]	INF β-1b s.c. 8 MIU every other day vs placebo	Phase III 3 years	92 INF β-1b n = 48, placebo n = 44	SPMS	Not significant effect of treatment with INF β-1b on cerebral volume loss (p = 0.343, from baseline to 3 years) compared with placebo.
Kappos et al. [176, 177]	INF β-1βs.c. 250 μg every other day (early vs delayed treatment)	Extension study of the Phase III BENEFIT trial (3 and 5 years follow up)	Follow-up phase n = 418 early treatment n = 261, delayed treatment n = 157 5-year completers n = 358 early treatment n = 235, delayed treatment n = 123	CIS	Marginal, non-significant differences between early and delayed treatment (p = 0.15, from baseline to 3 years, p = 0.121 from baseline to 5 years)
Calabresi et al. [111]	Peginterferon b-1a s.c. 125 μg Q2 W vs Q4 W vs placebo	Phase III ADVANCE 1 year, then open label	1512 PEG-IFN β-1a 125 μg Q2 W n = 512, PEG-IFN β-1a 125 μg Q4 W n = 500, placebo n = 500	RRMS	Core study: During the first 6 months of treatment there was a significant “pseudoatrophy” effect (PEG-IFN β-1a 125 μg Q2 W vs placebo, p = 0.0170) Baseline to year 1: Νo significant differences on whole brain volume between groups (Q2Wvs placebo p = 0.0841; Q4 W vs placebo p = 0.3747)
Arnold et al. (F2069, 1rst EAN Congress 2015) [112]	Peginterferon b-1a s.c. 125 μg Q2 W vs Q4 W	Extension study of Phase III ADVANCE 2 years	At week 96/569 PEG-IFN β-1a 125 μg Q2 W n = 384, PEG-IFN β-1a 125 μg Q4 W n = 185 (delayed treatment)	RRMS	From week 24 to week 96, the delayed treatment PEG-IFN β-1a 125 μg Q4 W n = 185 demonstrated a significantly greater decrease in whole brain volume compared with the Q2 W group (p = 0.0034)
Sorensen et al. [110]	INF β-1a s.c. 44 μg plus Methylprednisolone orally 200 mg or placeboorally	Phase III NORMI.M.S 2 years	110 INF β-1a and oral methylprednisolone n = 54, IFN β-1a and placebo n = 56	RRMS	Mean changes in normalized brain parenchymal volume favored pulsed treatment with oral methylprednisolone combined with INF β-1a vs INFβ-1a monotherapy, but the benefit was not significant (p = 0.25) between baseline and week 96
Ravnborg et al. [109]	INF β-1a i.m. 30 μg once weekly plus Methylprednisoloneorally 500 mg daily (3 consecutive days per month for 3–4 years) or placebo	Phase III MECOMBIN 3 years	338 INF β-1a plus placebo n = 167, INF β-1a plus methylprednisolone n = 171	RRMS	The study showed no effect on brain parenchymal volume (p = 0.58) or change in normalized brain volume (p = 0.52)
Comi et al. [114]	GAs.c. 20 mg daily vs Placebo	Phase III PreCISe 2 years	481 GA n = 243, Placebo n = 238	CIS	No significant difference in percentage change from baseline to last observed value in brain volume between the treatment groups (− 0.33% in GA vs − 0.38% in placebo)
Comi et al. [115]	GAs.c. 20 mgdailyvs placebo	open-label, extension phase of Phase III PreCISe 2 years	409 early-treatment group n = 198, placebo (delayed-treatment) n = 211	CIS	Significant reduction of BVL in early versus delayed treatment onset (28% reduction, p = 0.0209)
Ge et al. [116]	GAs.c. 20 mg daily vs placebo	Phase III The US GA study 2 years	27 GA treated n = 14, placebo n = 13	RRMS	GA significantly reduced the rate of BVL (77% reduction) in the 2-year treatment period (p = 0.007) compared with placebo
Rovaris et al. [119]	GA s.c. 20 mg daily vs placebo for 9 months, then GA open-label	Phase III European/ Canadian GA trial 18 months	227 GA n = 113, placebo n = 114	RRMS	During the double-blind, placebo-controlled phase of the study, GA treatment did not have any measurable impact on the absolute or percentage change of BV (from baseline to 9 months, p = 0.88) In the subsequent open-label phase, early GA treatment showed a 40% reduction in the rate of brain atrophy (from 9th to 18th month)
Rovaris et al. [118]	GA s.c. 20 mg daily	Extension of the Phase III European/ Canadian GA trial 5 years	142 Early treatment n = 73 Delayed treatment n = 69	RRMS	Baseline to 5 years: Non-significant differences in median PBVC in early vs delayed treatment groups.
Comi et al. [113]	GA s.c. 20 mg vs 40 mg (dose comparison)	Phase III FORTE 1 year	1155 GA 20 mg n = 586, GA 40 mg n = 569	RRMS	PBVCs were similar in both groups (p = 0.423). Higher dose of GA did not have a clear-cut impact on brain volume loss. Slower atrophy rates, compared with the Eur/Canadian GA trial
Khan et al. [178]	GA s.c. 40 mg TIW vs placebo	Phase III GALA 1 year	1263 GA 40 mg TIW n = 840, placebo n = 423	RRMS	The percentage change in brain volume (from baseline to 1 year) was not statistically different between treatment arms (− 0.706 with GA vs − 0.645 with placebo; p = 0.2058)
Lublin et al. [179]	INF β-1a i.m. 30 mg weekly, GA s.c. 20 mg daily	Phase III CombiRx 3 years	790 IFN + GA n = 388, IFN n = 187, GA n = 215	RRMS	Combination treatment was not superior to either INF β-1a or GA agents alone (CSF volume change from baseline to month 36; IFN β-1a + GA vs IFN, p = 0.008, INF β-1a vs GA p = 0.48). Whole brain tissue loss was reflected by the change in normalized CSF from baseline
O’Connor et al. [180]	INF β- 1b s.c. 250 μg or 500 μg, every other day or GA s.c. 20 mg daily	Phase III BEYOND 2 years	2244 IFN β-1b 500 μg n = 899, IFN β-1b 250 μg n = 897, GA n = 448	RRMS	Non-significant differences between treatment groups High dose INF β-1b was not superior to the standard dose (500 μg IFN β-1b vs 250 μg IFN β-1b p = 0.74). Both doses of IFN β-1b had similar measurable brain volume (BV) effect as compared with GA (500 μg IFN β-1b vs GA p = 0.33; 250 μg IFN β-1b vs GA p = 0.46). During year 1, patients under IFN β-1b had a significantly greater reduction in mean brain volume than did patients treated with GA (250 μg IFN β-1b vs GA p = 0.02; 500 μg IFN β-1b vs GA p = 0.007)
Arnold et al. [136]	DMF orally 240 mg BID vs TID vs placebo	Phase III DEFINE 2 years	540 DMF BID n = 176, DMF TID n = 184, Placebo n = 180	RRMS	Significant results for the DMF BID versus placebo on brain atrophy, from either baseline or 6 months to second year (baseline to 2 years p = 0.0449, 6 months to 2 years p = 0.0214). Non-statistically results for the DMF TID dose group
Miller et al. [137]	DMF orally 240 mg BID vs TID vs GA 20 mg once daily vs placebo	Phase III CONFIRM 2 years	681 DMF BID n = 169, DMF TID n = 170, GA n = 175, placebo n = 167	RRMS	At 2 years, PBVC favored DMF BID, but not TID or GA, compared to placebo (BID vs placebo; p = 0.0645; TID vs placebo; p = 0.2636; GA vs placebo p = 0.8802)
Kappos et al. (P7.243, AAN) [181]	DMF orally 240 mg BID vs TID vs placebo	8 year follow-up study of Phase III ENDORSE Ongoing	year 1/464 DMF BID n = 197, GA n = 88, placebo n = 179	RRMS	There was no significant effect in brain volume loss for the placebo/DMF and the GA/DMF groups relative to the group treated continuously with DMF BID (BID/BID group) (median PVC, from baseline to 5 years, BID/BID vs placebo/DMF p = 0.1165, BID/BID vs GA/DMF p = 0.3436)
Miller et al. [129]	Teriflunomide orally 7 or 14 mg once-daily vs placebo	Phase III TOPIC 4 years	614 Teriflunomide 14 mg n = 214, Teriflunomide 7 mg n = 203, placebo n = 197	CIS	No significant differences were recorded for brain atrophy (SIENA). (Mean change from baseline at week 108 vs placebo, 14 mg p = 0.4495; 7 mg p = 0.4462)
O’Connor et al. [130]	Teriflunomide orally 7 or 14 mg once-daily vs placebo	Phase III TEMSO 2 years	1086 Teriflunomide 14 mg n = 358, Teriflunomide 7 mg n = 365, placebo n = 363	RRMS	No effect on relative BPF change among the study groups (from baseline to 2 years: Teriflunomide 7 mg vs. placebo p = 0.19; Teriflunomide 14 mg vs. placebo p = 0.35)
Wolinsky et al. [131]	Teriflunomide orally 7 or 14 mg once-daily vs placebo	Post hoc analysis of Phase III TEMSO 108 weeks	1088 Teriflunomide 14 mg n = 359, Teriflunomide 7 mg n = 366, placebo n = 363	RRMS	There was a significant decrease in WM fraction (from baseline to108 weeks) for both doses of Teriflunomide (WMF change 14 mg vs placebo p = 0.0002; 7 mg vs placebo p = 0.0609)
Radue et al. (P3-089 AAN 2016) [134]	Teriflunomide orally 7 or 14 mg once-daily vs placebo	Post hoc analysis of Phase III TEMSO and TOWER 9 years	969 808 baseline and week 48, 709 baseline and week 108	RRMS	Significant gain in brain volume loss, by using an alternative method (SIENA). Median PVC, from baseline to first year, Teriflunomide 14 mg vs placebo p = 0.0001; Teriflunomide 7 mg vs placebo p = 0.0011; from baseline to second year: Teriflunomide 14 mg vs placebo p = 0.0001; Teriflunomide 7 mg vs placebo p = 0.0019)
Sprenger et al. P3.047 [132]	Teriflunomide orally 14 mg once-daily vs placebo	Post hoc analysis of Phase III TEMSO and TOWER 2 years	969 808 first year, 709 s year	RRMS	Teriflunomide resulted in lower atrophy rate in patients with and without disability progression vs placebo. Without disability progression: Median PBVC, from baseline to first year, Teriflunomide 14 mg vs placebo (22%) p = 0.0128 and from baseline to second year (23%) p = 0.0129 With disability progression: Median PBVC, from baseline to first year, Teriflunomide 14 mg vs placebo (69%) p = 0.0037 and from baseline to second year (44%) p = 0.0043
Wuerfel et al. P3.052 [135]	Teriflunomide orally 14 mg once-daily vs placebo	Post hoc analysis of Phase III of TEMSO	Year one cohort. 0–2 in previous 2 year: Teriflunomide 14 mg n = 191, placebo n = 197 2–3 in previous 2 year Teriflunomide 14 mg n = 195, placebo n = 198	RRMS	Significant impact on median PBVC regardless of the level of disease activity (prior relapse rate) Patients with few prior relapses (0–2 in previous 2 years): Baseline to year 1: Teriflunomide 14 mg vs placebo, relative change in percentage brain volume 40% p = 0.0001. Year 1to year 2: relative change 36%, p = 0.0001. This finding was confirmed in patients with a greater number of relapses (2–3 in previous 2 years): p = 0.0018 at year 1 and p = 0.0067 at year 2
Freedman et al. (P734 ETCRIMS 2016) [133]	Teriflunomide orally 7 or 14 mg once-daily vs placebo	Subgroup analysis of Phase III TEMSO	971 treatment-naïve n = 704, 1 Prior DMT n = 208, ≥2 Prior DMTs n = 57	RMS	Positive results on median PBVC regardless of treatment history. PVC change from baseline to year 1, Teriflunomide 14 mg No prior DMT vs placebo p = 0.0025; baseline to year 2: p = 0.0109; Teriflunomide 14 mg prior DMT vs placebo p = 0.0119, baseline to year 2: p = 0.0109. PVC change from baseline to year 1: Terilunomide 7 mg No prior DMT vs placebo p = 0.0002; baseline to year e: p = 0.0089. Teriflunomide 7 mg prior DMT vs placebo p = 0.0119, baseline to year 2: p = 0.0109

mg: milligrams; mcg: micrograms; μg: micrograms; vs: versus; PBVC: percentage of brain volume change; BPF: brain parenchymal fraction; BPV: brain parenchymal volume; SIENA: structural imaging evaluation using normalization of atrophy; i.m.: intramuscular; s.c: subcutaneous; i.v.: intravenous; ΤΙW: three times weekly; SD: standard deviation;Q2W: once every 2 weeks; Q4W: once every 4 weeks; BID: twice daily; TID: thrice daily; DMT: disease modifying therapies; CSF: cerebrospinal fluid; PVC: percentage volume change

**Table 2:** Second line therapies and their effect on brain volume loss (BVL)

References	DMT and trial design	Clinical trial	Baseline/MRI cohorts	Type of MS	Main effect on brain atrophy
Kappos et al. [122]	Fingolimod orally 0.5 mg or 1.25 mg once daily vs placebo for 2 years, then FTY open-label	Phase III FREEDOMS 2 years	1272 Fingolimod 1.25 mg n = 429, Fingolimod 0.5 mg n = 425, placebo n = 418	RRMS	Significant reductions in the rate of brain volume loss were detected as early as 6 months for the 12 mg Fingolimod treatment group (PBVC values from baseline to 6 months, 1.25 mg Fingolimodvs placebo p = 0.003; 0.5 mg Fingolimodvs placebo p = 0.006) and remained significant at 24 months (P < 0.001 in all comparisons)
Kappos et al. [124]	Fingolimod orally 0.5 mg or 1.25 mg once daily (FTY open label)	Extension of Phase III FREEDOMS 2 years	920	RRMS	Significantly lower atrophy rates in the continuous Fingolimod groups relative to the combined switch group, over 4 years (Continuous Fingolimod 0.5 mg p = 0.0013; Continuous Fingolimod 1.25 mg p = 0.001) Patients who switched to Fingolimod 0.5 mg during the extension study experienced significant improvements in rates of brain volume decline (Placebo—Fingolimod 0.5 mg p = 0.0084, months 24–48 vs months 0–24)
Cohen et al. [121]	Fingolimod orally 1.25 or 0.5 mgonce daily vs INF β-1a i.m. 30 μg (1 year, then open-label)	Phase III TRANSFORMS 1 year	1280 Fingolimod 1.25 mg n = 420, Fingolimod 0.5 mg n = 429, INF β-1a N = 431	RRMS	Compared to i.m. INF β-1a, patients treated with Fingolimod presented less brain volume loss, over 1 year (all p < 0.001)
Khatri et al. [125]	Fingolimod orally 1.25 or 0.5 mg once daily	Extension of Phase III TRANSFORMS 2 years	799 INF β-1a to 0.5 mg Fingolimod n = 124, INF β-1a to 1.25 mg Fingolimod n = 130. Continuous 0.5 mg Fingolimod n = 290, Continuous 1.25 mg Fingolimod n = 255	RRMS	Patients switching from INF β-1a to Fingolimod (either 1.25 or 0.5 mg) reduced their brain volume decrease (PBVC: months 13–24 vs months 0–12, p = 0.006 for the INF β-1a to 0.5 mg Fingolimod switch group p = 0·007 for the INF β-1a to 1.25 mg FTY720 switch group. No further gain in BVL for patients on continuous Fingolimod treatment (p values non-significant at months 13–24 vs months 0–12)
Calabresi et al. [120]	Fingolimod orally 1.25 or 0.5 mg once daily vs placebo	Phase III FREEDOMS II 1 year	1083 Fingolimod 1.25 mg n = 370, Fingolimod 0.5 mg n = 358, placebo n = 355	RRMS	Patients given Fingolimod had decreased brain volume loss compared with those given placebo from baseline to months 6 (Fingolimod 1.25 mg vs placebo, p = <0.0001; Fingolimod 0.50 mg vs placebo, p = 0.012) 12 (Fingolimod 1.25 mg vs placebo, p = <0.0001; Fingolimod 0.50 mg vs placebo, p = 0.004) and 24 (Fingolimod 1.25 mg vs placebo, p = <0.0001; Fingolimod 0.50 mg vs placebo, p = 0.013). (Total treatment effect on PBVC vs placebo p < 0·0001 and p = 0·0002 respectively)
Cohen et al. [123]	Fingolimod orally 1.25 or 0.5 mg once daily IFN β-1a i.m. 30 μg once a week	Extension of Phase III TRANSFORMS 4.5 years	Fingolimod 0.5 mg n = 356, IFN β-1a-switch Fingolimod 0.5 mg n = 167, Fingolimod 1.25 mg n = 330, IFN β-1a switch fingolimod 1.25 mg n = 174	RRMS	Non-significant long term benefit on mean PBVC (from baseline to 4.5 years): continuous-fingolimodvs IFN β-1a-switch group −1.01% (−0.8) vs −0.96% (−0.8); p = 0.937. The PBVC from baseline to month 12 was reduced significantly by fingolimod compare to IFN β-1a (p < 0.0001) and the low rate was maintained through the study completion
Lublin et al. [126]	Fingolimod orally 0.5 mg once daily vs placebo	Phase III INFORMS 3 years	714 Fingolimod 0.5 mg n = 293, placebo n = 421	PPMS	In patients with primary progressive MS, percentage change in brain volume did not differ between Fingolimod and placebo groups (p = 0.673)
Miller et al. [139]	Natalizumab i.v. 300 mg every 4 weeks vs placebo	Phase III AFFIRM 2 years	942 Natalizumab n = 627, placebo n = 315	RRMS	Overall, not significant effect of treatment with Natalizumabvs placebo (mean percentage change in BPF, 0.80% vs 0.82%, p = 0.822, from baseline to 2 years). During the first year, natalizumab-treated patients presented greater BVL compared to placebo (0.56% vs 0.40%, p = 0.002) but the rate of brain atrophy was significantly less in natalizumab-treated patients over the second year of treatment (0.24% vs 0.43% p = 0.004)
Radue et al. [140]	(IFN β-1a i.m.30 μg + Natalizumab i.v.300 mg every 4 weeks) vs IFN β-1a i.m. 30 μg + placebo once weekly	Phase III SENTINEL 2 years	1171 IFN β-1a + natalizumab n = 589, IFN β-1a + placebo n = 582	RRMS	From baseline to second year, no significant differences were reported between the 2 treatment arms regarding change in BPF (p = 0.926). During the first year, there was a significant reduction in BPF in the Natalizumab treated arm (p = 0.058), but lower rates during the 2nd year of treatment (– 0.31% versus – 0.40%; p = 0.020)

mg: milligrams; mcg: micrograms; μg: micrograms; vs: versus; PBVC: percentage of brain volume change; BPF: brain parenchymal fraction; BPV: brain parenchymal volume; SIENA: structural imaging evaluation using normalization of atrophy; i.m.: intramuscular; s.c: subcutaneous; i.v.: intravenous; ΤΙW: three times weekly; SD: standard deviation;Q2W: once every 2 weeks; Q4W: once every 4 weeks; BID: twice daily; TID: thrice daily; DMT: disease modifying therapies; CSF: cerebrospinal fluid; PVC: percentage volume change

**Table 3:** New or emerging therapies and their effect on brain volume loss (BVL)

References	DMT and trial design	Clinical trial	Baseline/MRI cohorts	Type of MS	Main effect on brain atrophy
Comi et al. [157]	Laquinimod orally 0.6 mg once daily vs placebo	Phase III ALLEGRO 2 years	1106 Laquinimod n = 550, placebo n = 556	RRMS	Laquinimod had a significant effect on reducing brain volume loss vs placebo (p < 0.001, from baseline to 2 years)
Vollmer et al. [158]	Laquinimod orally 0.6 mg once daily vs IFN β-1a i.m. 30 μg once weekly vs oral placebo	Phase III BRAVO 1 year	1331 Laquinimod n = 434, IFN β-1a i.m. n = 4 47, placebo n = 450	RRMS	Robust effects on reducing brain atrophy are replicated for Laquinimod (p < 0.001, from baseline to year 1), whereas IFN β-1a showed no benefit at all (non-significant. increased BVL 11% vs placebo, p = 0.14)
Cohen et al. [147]	Alemtuzumab i.v. 12 mg (once per day for 5 days at baseline and once per day for 3 days at 12 months) vs INF β-1a s.c. 44 μg TIW	Phase III CARE-MS I 2 years	563 Alemtuzumab n = 376, INF β-1a n = 187	RRMS	Median change in brain parenchymal fraction was less in Alemtuzumab (− 0.867%) was compared with INF β-1a (1.488%), p < 0.001)
Coles et al. [148]	Alemtuzumab i.v. 12 mg once per day vs 24 mg once per day (once per day for 5 days at baseline and for 3 days at 12 months) vs INF β-1a s.c. 44 μg TIW	Phase III CARE-MS II 2 years	628 Alemtuzumab 12 mg n = 426, INF β-1a n = 202	RRMS	Compared to 44 μgsc IFN β-1a (− 0.810%), alemtuzumab-treated (− 0.615%) patients showed less reduction in median parenchymal brain fraction during the first year of the trial (p = 0.01)
Traboulsee et al. P1181 ECTRI.M.S [6]	Alemtuzumab i.v. 12 mg once daily received 2 annual courses (on 5 consecutive days at baseline and on 3 consecutive days 12 months later). Patients could receive additional treatment with alemtuzumab (12 mg on 3 consecutive days ≥ 1 year after the most recent course) during the extension study	Extension of Phase III CARE-MS I, CARE-MS II 4 years	93% of CARE-MS I n = 325, 88% of CARE-MS II n = 393	RRMS	Durable MRI positive outcomes (i.e. sustained low brain atrophy rates, in the absence of continuous treatment with Alemtuzumab or other DMTs during the follow up period)
Coles et al. [150]	Alemtuzumab i.v.(12 mg on 3 consecutive days) Alemtuzumab-treated patients who completed CARE-MS II could enroll in the extension and receive, at the investigator’s discretion, additional alemtuzumab courses (12 mg on 3 consecutive days) ≥ weeks after the most recent course, if they had evidence of MS disease activity. Patients who received s.c. IFN-b-1a for 2 years in the core study could also enroll in the extension and switch to alemtuzumab treatment; results for these patients will be reported separately	CARE-MS II 5 years follow-up	Most alemtuzumab-treated patients (92.9%) who completed CARE-MS II entered the extension; 59.8% received no alemtuzumab retreatment	RRMS	Median yearly BVL remained low in the extension (years 1–5: − 0.48%, − 0.22%, − 0.10%, − 0.19%, − 0.07%). Yearly BVL rate continued to decrease in year 3 compared with the core study, remaining low in years 4 and 5. Median BPF change from baseline to year 5 was − 0.855%
Arnold et al. (P558, ECTRI.M.S 2015) [151]	Daclizumab s.c 150 mg every 4 weeks vs INF β-1ai.m. 30mcg once weekly	Post hoc of Phase III DECIDE 2 years	1806 Daclizumab n = 899, INF β-1a n = 907	RRMS	Daclizumab showed a significant effect in limiting the rate of brain atrophy vs IFN β-1a, between baseline and week 96 (p < 0.0001), week 0 and week 24 (p = 0.0325) and between week 24 and week 96 (p < 0.0001)
Montalban et al. [155]	Ocrelizumab i.v. 600 mg (two 300 mg infusions 14 days apart) vs placebo	Phase III ORATORIO	732 Ocrelizumab 600 mg, n = 488, placebo n = 244	PPMS	Ocrelizumab reduced the rate of whole brain volume loss from week 24 to week 120 by 17.5%120 (p = 0.0206) compared with placebo
Arnold et al. [154]	Ocrelizumab i.v 600 mg.every 24 weeks vs INF β-1as.c. 44 mcg TIW	Phase III OPERA I 96 weeks	821 Ocrelizumab n = 410, IFN β-1a n = 411	RMS	Ocrelizumab reduced brain volume loss compared with INF β-1a. (p < 0.001 from baseline to 96th week and p = 0.0042 from 24th to 96th week)
Arnold et al. [154]	Ocrelizumab i.v 600 mg.every 24 weeks vs INF β-1as.c. 44 mcg TIW	Phase III OPERA II	835 Ocrelizumab n = 417, IFN β-1a n = 418	RMS	Ocrelizumab reduced brain volume loss compared with INF β-1a. [p = 0.001 from baseline to 96th week and p = 0.09 (non-significant) from 24th to 96th week]
De Stefano et al. [162]	Cladribine3.5 mg/kg or Cladribine5.25 mg/kgvs placebo	Phase III CLARITY	1025 Cladribine 3.5 mg/kg n = 336, Cladribine 5.25 mg/kg n = 351, placebo n = 338	RMS	Patients treated with cladribine had significantly less annualized brain atrophy over 2 years compared with patients receiving placebo. At 18 months, patients treated with cladribine had 20% reduction in brain atrophy compared with patients receiving placeboIn patients under cladribine tablets 3.5 mg/kg (− 0.56% ± 0.68, p = 0.010) and 5.25 mg/kg (− 0.57% ± 0.72, p = 0.019), the annualized PBVC was reduced compared with placebo (− 0.70% ± 0.79)

mg: milligrams; mcg: micrograms; μg: micrograms; vs: versus; PBVC: percentage of brain volume change; BPF: brain parenchymal fraction; BPV: brain parenchymal volume; SIENA: structural imaging evaluation using normalization of atrophy; i.m.: intramuscular; s.c: subcutaneous; i.v.: intravenous; ΤΙW: three times weekly; SD: standard deviation;Q2W: once every 2 weeks; Q4W: once every 4 weeks; BID: twice daily; TID: thrice daily; DMT: disease modifying therapies; CSF: cerebrospinal fluid; PVC: percentage volume change

In general, studies of traditional injectable treatments have not exerted robust beneficial effects in the rate of brain atrophy. Intramuscular IFN-β-1a produced lower rates of brain volume loss (BVL) when compared to placebo during the second year of treatment in relapsing–remitting MS patients (− 0.23% vs − 0.51%; p = 0.03) [83, 104]. However, the subcutaneous (sc) IFN-β-1a produced inconsistent results in both CIS and RRMS patients [105–108]. BV data for intramuscular INF-β-1a in CIS patients and for subcutaneous INF-β-1b in relapsing MS patients has not been made available to date. The addition of monthly oral methylprednisolone pulses to subcutaneous interferon beta-1a treatment provided no further gain in normalized BV change in two published trials against placebo [109, 110]. The approved long-acting pegylated interferon beta-1a has only shown limited and inconclusive evidence for a beneficial effect on BV change in RRMS [111, 112]. A possible delayed effect in reducing brain atrophy has been reported for Glatiramer acetate [GA] [113–119]. In the PReCISe clinical trial, GA failed to show an immediate effect on brain volume outcomes versus placebo (− 0.38% vs 0.33%), but the subsequent open label phase of the trial showed a clear–cut benefit on PBCV for the early treatment group, when compared to patients with delayed treatment onset (40% reduction, p = 0.0209) [114, 115]. In relapsing–remitting MS, data from the extension phase of the European/Canadian GA trial come back as negative [118].

Available oral therapies (Fingolimod, Teriflunomide, Dimethyl fumarate) have shown various effects on BV decline. Fingolimod has been reported consistentin reducing median PBVC by approximately 30 to 45% versus placebo or IFNβ-1a, in its three phase III clinical trials [120–122] and their extensions [123–125]. Of note, this reduction was observed as early as 6 months after treatment onset [120, 122]. In the extension phase of the TRANFORMS trial, patients switching from intramuscular (i.m.) INF β1a to FTY720 slowed their median PBVC, and patients continuing on FTY720 sustained low atrophy rates, over the following 4.5 years of therapy [123]. However, no similar effects were reproduced in patients with the primary progressive form of MS, a finding that that could have otherwise strengthened the evidence for a direct action of fingolimod on brain cellular components [126]. Finally, further condoning the aforementioned observations, in a study by Yousuf et al. [127], cortical GM, alongside T2 lesion volume, remained stable in the cohort treated with fingolimod, as compared to the untreated group, where it decreased and increased respectively, in the first 2 years of treatment.

Regarding Teriflunomide [128], brain volume outcomes have been reported for clinically isolated syndrome and relapsing- remitting MS in the TOPIC and TEMSO clinical trials respectively. Both doses of 7 mg or 14 mg failed to show a clear effect on slowing BVL when compared to placebo [129, 130]. However, when tissue specific volume changes were examined a significant reduction in the rate of WM loss was detected for the 14 mg teriflunomide treatment arm versus placebo [131]. Similar results have recently been reported in 4 retrospective analyses of TOWER and TEMSO trials when an alternative method of brain loss evaluation was implemented [132–135].

Dimethyl fumarate (DMF/BG12) showed a 21% reduction in BVL compared to placebo in the DEFINE study (the 240 mg twice daily regimen only) [136] and produced only marginal but beneficial effects in BVL reduction in the CONFIRM study [137]. A recent pilot study of 20 patients with RRMS showed a protective effect of DMF treatment in whole brain atrophy (PBVC: − 0.37 ± 0.49% vs. − 1.04 ± 0.67%, p = 0.005) and putamen atrophy (− 0.06 ± 0.22 vs. − 0.32 ± 0.28 ml, p = 0.02), but no effect on other subcortical volumes or total GM atrophy [138].

Natalizumab, a monoclonal antibody against the cell adhesion molecule a 4-integrin, in two pivotal clinical trials was found to increase the rate of BVL in the first year of treatment and then significantly reduced it when compared to the placebo in the second year [139, 140]. Post–marketing observational studies confirmed that most of the BVL occurring while on Natalizumabtherapy takes place during the first months of therapy, and that it primarily involves WM volume changes [141, 142]. One trial has shown superiority of Natalizumab over conventional MS therapies (IFN-β and GA) and placebo regarding cortical atrophy [143]. Recently, treatment with Natalizumabdid not affect the loss of brain volume compared to placebo in secondary progressive MS patients (ASCENT) [144]. Τhe study by Arpín et al. [145] also suggests a neuroprotective effect of Natalizumab, after the measurement and comparison of the corpus calosum index, and the absence of brain atrophy in several patients under treatment during the follow up.

Alemtuzumab, a monoclonal antibody against cells that express the CD52, has demonstrated greater MRI and clinical improvement in comparison to IFNb-1a in its three pivotal studies in active relapsing MS patients [146–148]. Additionally, most patients remained free of disability and MRI progression, for the following 6 years of the initial treatment [6]. Brain atrophy measures showed that brain parenchymal fraction was smaller in Alemtuzumab compared to the INF β-1a treatment arm either in treatment naïve patients [149] or in participants who had relapsed on prior therapy [147–149]. Extension studies showed sustained low brain atrophy rates, in the absence of continuous treatment with Alemtuzumab or other DMTs during the follow up period [149]. The CARE-MS II 5-year follow-up study (2017) provided class III evidence that Alemtuzumab slows brain atrophy; the annual BVL rate continued to drop during the third year and remained low through the fourth and fifth year as well [150].

The immune-modulatory agent Daclizumab in a 3-year post hoc analysis of 899 RRMS patients was compared to IFN beta-1a on brain volume change. Median annualised PBVC was significantly reduced in the DAC treatment group during both the first and the second year of treatment (baseline—24th weeks: − 0.674 vs − 0.745; 24th–96th weeks: − 0.511 vs − 0.549; all p < 0.0001) in comparison to INF β treatment [151], a finding which was consistent with previous longitudinal data [151–153].

Ocrelizumab is a humanized mAb designed to target CD20+ B cells. MRI outcomes hint towards a positive effect on BVL and clinical disability progression. Treatment with Ocrelizumab has significantly slowed brain atrophy rates in comparison to INF-β1a (baseline to 96 weeks: 23.5% p < 0.0001 in OPERA 1 and 23.8% p < 0.0001 in OPERA 2) along with clinical disability [154]. Ocrelizumab reduced the rate of whole BVL in PPMS from week 24 to week 120 by 17.5%120 (p = 0.0206) compared with placebo (ORATORIO) [155].

### Emerging DMTs and their effect on PBVC

Several new agents are currently undergoing clinical development, including immuno-modulatory, neuroprotective or remyelinating compounds.

Laquinimod, a linomide derivative, has also shown promising results on PVC rates in RRMS, most probably as a result of reduced astrocytic activation within the CNS [156]. In the ALLEGRO clinical trial, after adjusting for baseline active inflammation, laquinimod markedly reduced BVL as compared to the placebo [157]. Positive effects on PBVC are replicated in one active comparator trial [BRAVO] versus im IFN-β-1a [158, 159]. At present, the agent is further investigated in RRMS [CONCERTO] and PPMS patients [ARPEGGIO].

Cladribine, an antiproliferative agent that takes effect by interfering with DNA synthesis, has shown significant effects in terms of relapse rate and disability progression [160, 161]. Data from CLARITY study suggested that at 18 months, patients treated with cladribine had 20%reduction in brain atrophy compared with patients receiving placebo [162]. However, further studies are needed, in order to cladribine’s effect on brain atrophy rates, be fully elucidated [161, 163, 164].

## Conclusions

MS is an evolving disease, now considered of both inflammatory and neurodegenerative nature [165–168]. Axonal injury and loss accounting for brain atrophy may be either acute (i.e. due to inflammation) or chronic/late due to pathogenic mechanisms primed by the preceding inflammation and later perpetuating with disease progression [169–171]. Brain atrophy occurs as early as CIS, progresses faster than it does in healthy adults, and is the best predictor of future disability, physical and cognitive [166, 172]. It is widely accepted to be a valid, sensitive and reproducible measure of neuroprotection in MS research studies and therapeutic trials.

There is now a variety of approved DMDs, with secondary neuroprotective properties, and an even greater number of novel compounds, in various stages of development and investigation. A firm belief remains that for a therapy to be effective in delaying the disease progression, its impact on axon and neuronal survival needs to be monitored. Conventional MRI findings (T1-hypotensive or T2 hypertensive lesion load) have already shown their limits for monitoring the disease burden and progression in MS patients. Newly introduced sophisticated imaging methods hold promise for the future of the clinical surveillance of the disease. Trials incorporating brain atrophy in their endpoints are providing accumulating evidence that rises substantial hopes for treating neurodegeneration in the near future.
